# Evaluating the accuracy of ultrasonography in the diagnosis of subtle Lisfranc injuries

**DOI:** 10.1002/jeo2.70434

**Published:** 2025-09-09

**Authors:** Kensei Yoshimoto, Toru Omodani, Kotaro Ishizuka, Kazunori Maruyama, Mitsuki Kumaki, Masahiko Noguchi, Ken Okazaki

**Affiliations:** ^1^ Department of Orthopedic Surgery Tokyo Women's Medical University Shinjuku‐ku Tokyo Japan; ^2^ Orthopaedic Foot and Ankle Center Shiseikai Daini Hospital Setagaya‐ku Tokyo Japan; ^3^ Tokyo Advanced Orthopaedics Chofu Tokyo Japan; ^4^ Urasoe General Hospital Sports Medical Center Urasoe Okinawa Japan; ^5^ Rokuto Orthopaedic Clinic Urasoe Okinawa Japan

**Keywords:** diagnosis, Lisfranc ligament, subtle Lisfranc injuries, ultrasonography, ultrasound

## Abstract

**Purpose:**

Weight‐bearing (WB) computed tomography or plain radiography provides the most accurate diagnosis of subtle Lisfranc injuries. However, WB is often challenging for patients due to pain, and these modalities can be inconvenient. Recently, the utility of ultrasonography (US), which enables easy and convenient assessment and bilateral comparison, has gained attention. This study aimed to assess whether US can accurately diagnose these injuries.

**Methods:**

Twenty‐five patients with subtle Lisfranc injuries, defined as a > 2 mm distance between the first cuneiform (C1) and second metatarsal (M2) on WB anteroposterior plain foot radiographs, were included in this cross‐sectional study. Bilateral foot radiographs were used for intra‐individual comparison, and the contralateral side was confirmed to be uninjured. US without stress or WB was performed after radiography, and the C1–M2 dorsal and articular distances were measured to assess whether this modality could accurately diagnose subtle Lisfranc injuries. Patients with uninjured contralateral feet were evaluated as healthy subjects. All patients had ligament injury and instability confirmed intraoperatively.

**Results:**

US measurements demonstrated strong reliability: the intraclass correlation coefficients for the C1–M2 dorsal distance were 0.91 (intra‐observer) and 0.92 (inter‐observer), and for the articular distance, 0.95 and 0.94, respectively. The C1–M2 dorsal and articular distances were significantly greater on the injured side. The cutoff values for the C1–M2 dorsal and articular distances in diagnosing subtle Lisfranc injury were 9.3 mm and 2.1 mm, respectively. The sensitivity and specificity of the cutoff value for the C1–M2 dorsal distance were 0.88 and 0.72, respectively, whereas those of the cutoff value for the C1–M2 articular distance were 0.76 and 0.96, respectively.

**Conclusion:**

Although US examination requires experience, it demonstrated high diagnostic accuracy in detecting subtle Lisfranc injuries without the need for stress or WB imaging and showed high consistency in both intra‐ and inter‐observer measurements of the C1–M2 distance.

**Levels of Evidence:**

Level III.

**Abbreviations**
AUC: area under the curve
C1: first cuneiform
CI: confidential intervals
CT: computed tomography
ICCs: intraclass correlation coefficients
M2: second metatarsal
US: ultrasonography
WB: weight‐bearing

## INTRODUCTION

The term “Lisfranc injury” refers to ligamentous and osseous disruptions of the tarsometatarsal joint [15]. Epidemiologic studies have shown that although Lisfranc injuries account for only 0.2% of all fractures, it is estimated that over 20% remain undiagnosed [13]. These injuries are classified as either severe or subtle, depending on the underlying mechanism of trauma. Subtle injuries are typically associated with indirect, low‐energy mechanisms such as twisting forces or sprain‐type injuries [11, 20]. Although displaced injuries are easily identified, diagnosing those that can lead to subtle instability can be challenging because of their varying clinical presentations and radiographic findings [17, 20]. Because inadequately treated Lisfranc joint injuries can lead to significant morbidity and long‐term disability [4], accurate diagnosis is essential.

Subtle injuries are commonly evaluated by plain radiography [3, 17], with weight‐bearing (WB) imaging demonstrating higher sensitivity than non‐WB imaging [14, 18]. A previous study has shown that approximately 12% of such injury‐induced instability cases are also missed on WB radiographs; [9] thus, WB computed tomography (CT) can be particularly useful for evaluating subtle injuries [1, 9, 19, 21]. Since WB radiography and CT are not always available at all healthcare institutions and WB is often challenging for patients due to pain, a more convenient and painless diagnostic modality is needed.

Recently, the utility of ultrasonography (US) without ionising radiation that enables bilateral comparison has gained attention in the diagnosis of subtle Lisfranc injuries [5, 10]. Ghandour et al. [5] demonstrated in a cadaveric study that stress US can diagnose subtle Lisfranc injuries with a sensitivity of 81% and specificity of 72%. However, the clinical accuracy of US in detecting subtle Lisfranc injuries is still uncertain. Thus, this study aimed to evaluate its diagnostic accuracy in clinical practice. It was hypothesised that US would accurately identify subtle Lisfranc injuries.

## METHODS

### Subjects

**Figure 1:**
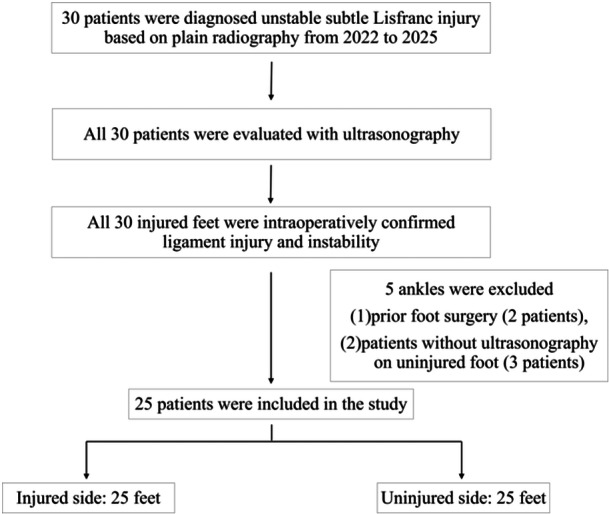
Flow diagram of the study.

This cross‐sectional study was approved by the institutional review board (IRB number: 148) and was conducted in accordance with the World Medical Association's Declaration of Helsinki. Thirty patients diagnosed with unstable subtle Lisfranc injury at two healthcare institutions from 2022 to 2025 were investigated. The criteria for the diagnosis of unstable subtle Lisfranc injury were as follows: (1) patients with a clear episode of trauma; (2) those with > 2 mm distance between the first cuneiform (C1) and second metatarsal (M2) on WB anteroposterior plain foot radiography; [15] and (3) patients without definite dislocation or fractures of the Lisfranc joint, with the exception of the fleck sign. Bilateral foot radiographs were used for intra‐individual comparison, and the contralateral side was confirmed to be uninjured. US without stress or WB was performed after radiography to assess whether this modality can accurately diagnose subtle Lisfranc injuries. Patients with uninjured contralateral feet were evaluated as healthy subjects. All patients had ligament injury and instability confirmed intraoperatively. The exclusion criteria were as follows: (1) prior foot surgery, and (2) patients without US on the uninjured foot. Ultimately, 25 patients were included in the analysis. Patients with uninjured contralateral feet were regarded as healthy subjects (Figure 1).

### Ultrasonographic evaluation

**Figure 2:**
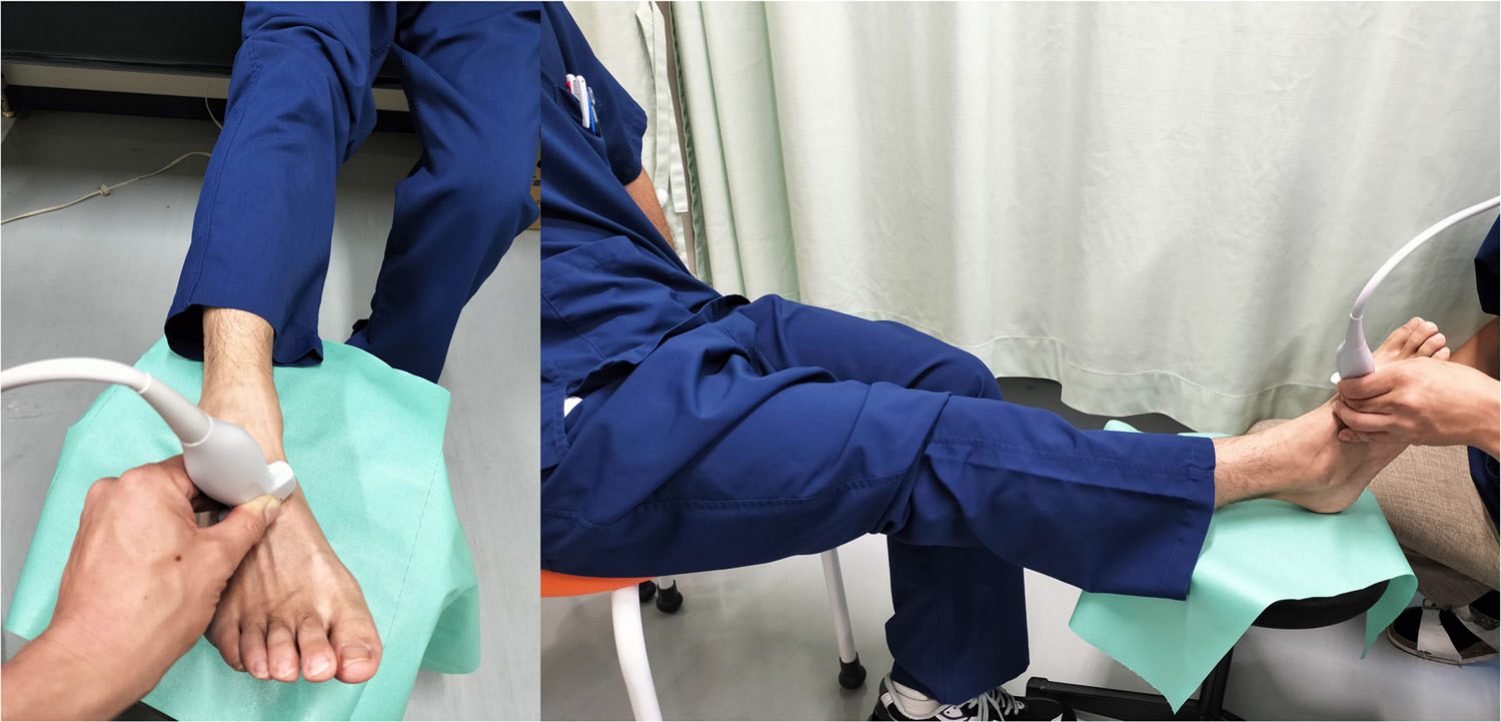
The ultrasound probe was placed on the dorsum of the C1 and M2 while the patients were in a sitting position, with hips flexed, knees extended, and ankles naturally plantar‐flexed.

**Figure 3:**
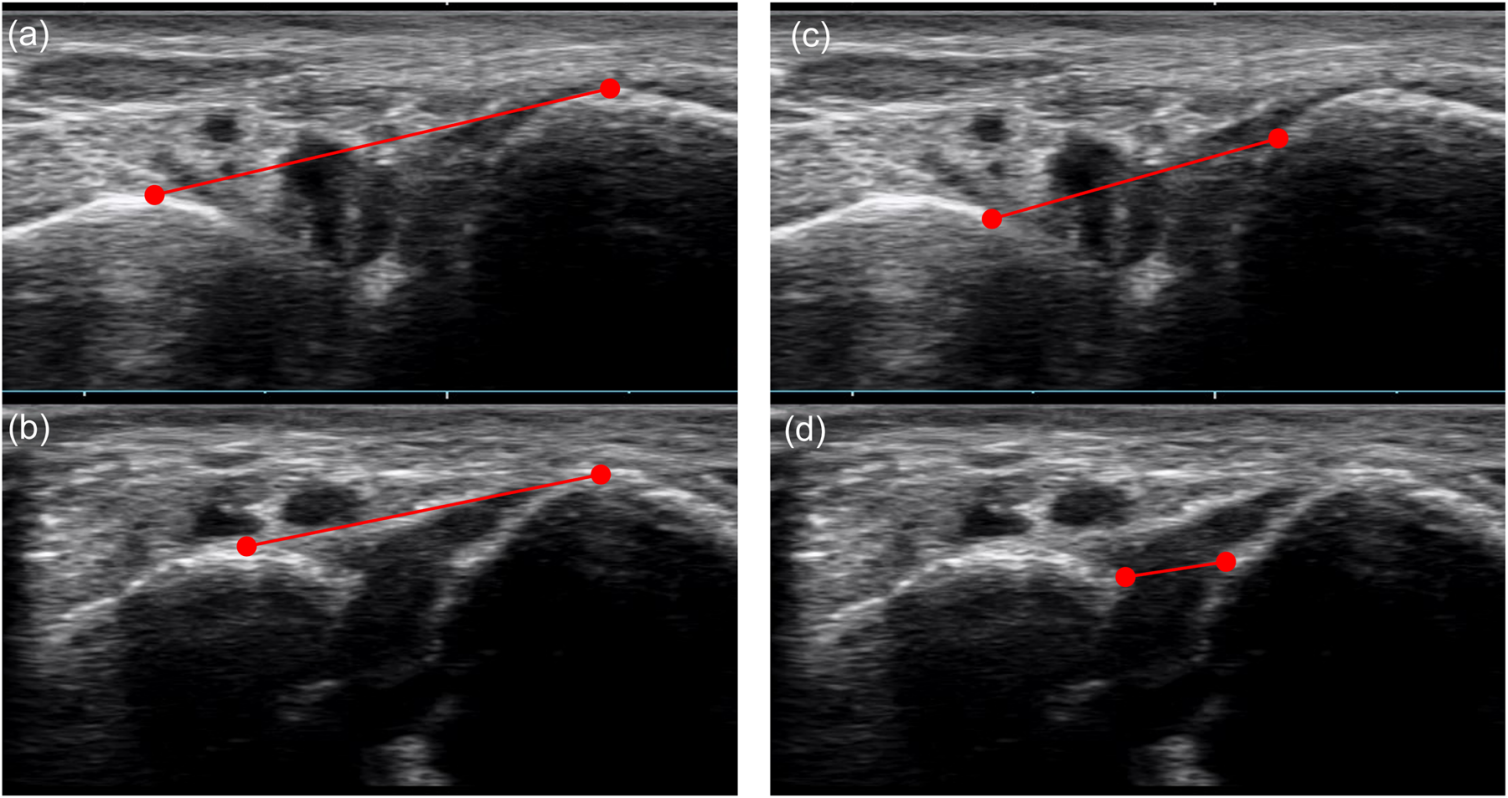
The C1–M2 dorsal distance was defined as the distance between the dorsal apex of C1 and M2. Injured (a) and uninjured (b) sides. The C1–M2 articular distance was defined as the distance between the innermost visible articular edges of the medial cuneiform and second metatarsal bone. Injured (c) and uninjured (d) sides.

US was performed by each attending surgeon using a SNIMAGE HS2 PRO (KONICA MINOLTA JAPAN, Tokyo, Japan) with a linear high‐frequency transducer (4–14 MHz) in the visualised B‐mode image display. The US examiners were not blinded because they could easily identify the injured side based on swelling and internal bleeding. The US probe was placed on the dorsum of the C1 and M2 while the patients were in a sitting position with hips flexed, knees extended, and ankles naturally plantar‐flexed, as this posture was easily assumed shortly after the injury (Figure 2). The C1–M2 dorsal and articular distances were then measured. The C1–M2 dorsal distance was defined as the distance between the dorsal apex of C1 and M2 (Figure 3a,b). The C1–M2 articular distance was defined as the distance between the innermost visible articular edges of C1 and M2 (Figure 3c,d) [5]. Two orthopedic surgeons who were blinded to the patients' records, measured the C1–M2 distance for both injured and uninjured sides, and one surgeon measured two times at 1‐month intervals.

### Statistical analysis

Statistical analysis was performed using JMP 14.0 software (SAS Institute, Cary, NC, USA). The intraclass correlation coefficients (ICCs) and their 95% confidential intervals (CI) were used to summarise the intra‐ and inter‐observer reproducibility of the radiographic measurements. The ICCs values were interpreted as follows: 1.0, perfect agreement; 0.81–0.99, excellent agreement; 0.61–0.80, good agreement; 0.41–0.60, moderate agreement; 0.21–0.40, fair agreement; 0.00–0.20, poor agreement. The data obtained by comparing the categorical and continuous variables between two groups were analysed using Fisher's exact and Mann–Whitney *U* test, respectively. *p*‐Values < 0.05 were considered statistically significant. The receiver operating characteristic curve analysis, based on the Youden index, was used to determine the cutoff value of the C1–M2 distance for subtle Lisfranc injury.

Power analysis, which was performed as described previously [5], indicated that a sample size of 22 patients would provide 80% statistical power for detecting a 0.5‐mm difference in C1–M2 distance on US.

## RESULTS

The 25 patients had a mean age of 29.0 ± 13.1 years at surgery (range, 14–52 years), with 13 men and 12 women. The mean body mass index was 23.1 ± 3.2 (range, 18.8–29.1).

The intra‐ and inter‐observer ICCs of the C1–M2 dorsal distance were 0.91 (95% CI, 0.77–0.96) and 0.92 (95% CI, 0.80–0.97), respectively. Those of the C1–M2 articular distance were 0.95 (95% CI, 0.88–0.98) and 0.94 (95% CI, 0.85–0.98), respectively.

The C1–M2 dorsal and articular distances were significantly longer on the injured side (10.7 ± 1.9 mm vs. 8.5 ± 1.7 mm, *p* < 0.0001; and 3.2 ± 1.5 mm vs. 1.1 ± 0.6 mm, *p* < 0.0001, respectively; Table 1). The cutoff values for the C1–M2 dorsal and articular distances for subtle Lisfranc injury were 9.3 mm and 2.1 mm, respectively. The sensitivity and specificity of the cutoff value for the C1–M2 dorsal distance were 0.88 (95% CI, 0.76–1.0) and 0.72 (95% CI, 0.58–0.94), respectively, whereas those for the C1–M2 articular distance were 0.76 (95% CI, 0.59–0.93) and 0.96 (95% CI, 0.94–0.98), respectively (Table 2). The 95% CIs of the differences between the uninjured and injured side were 1.5–2.9 mm for the C1–M2 dorsal distance and 1.5–2.7 mm for the C1–M2 articular distance.

**Table 1:** Comparison of C1–M2 distance on ultrasonography between injured and uninjured side.

	Injured (*n* = 25)	Uninjured (*n* = 25)	*p*‐Value
C1–M2 dorsal distance (mm)	10.7 ± 1.9	8.5 ± 1.7	<0.0001*
C1–M2 articular distance (mm)	3.2 ± 1.5	1.1 ± 0.6	<0.0001*

*Note*: Mean ± standard deviation.

* *p* < 0.05.

**Table 2:** The receiver operating characteristic curve analysis for determining optimal cut‐off values for subtle Lisfranc injuries.

	Cut‐off values (mm)	Sensitivity	Specificity	PPV	NPV	FPR	FNR	AUC	*p*‐Value
C1–M2 dorsal distance (mm)	9.3	0.88	0.72	0.79	0.86	0.24	0.12	0.81	0.001*
C1–M2 articular distance (mm)	2.1	0.76	0.96	0.95	0.80	0.04	0.24	0.89	0.001*

Abbreviations: AUC, area under the curve; FNR, false negative rate; FPR, false positive rate; NPV, negative predictive value; PPV, positive predictive value.

* *p* < 0.05.

## DISCUSSION

The most important finding of this study was the utility of US in accurately diagnosing Lisfranc injuries without stress or WB imaging, with the cutoff values for the C1–M2 dorsal and articular distances being 9.3 and 2.1 mm, respectively. Furthermore, differences > 1.5 mm in the C1–M2 dorsal and articular distances between the injured and uninjured sides could serve as another diagnostic criterion.

Previous studies have proposed definitions and measurements of Lisfranc injuries, mainly based on plain radiographs, CT, or magnetic resonance imaging (MRI) [3, 6, 18, 20]. Many studies recommend WB radiography or CT for more accurate evaluation of Lisfranc injuries [1, 2, 21, 22], even though non‐WB plain radiography or CT can also provide a diagnosis [2, 14, 16]. Because WB imaging is often difficult for patients with acute injury due to pain, the utility of performing US without WB for accurate diagnosis of subtle Lisfranc injuries, as identified in this study, is valuable. On the other hand, MRI allows for direct assessment of each component of the Lisfranc ligamentous complex. However, it is difficult to compare findings with the uninjured side using MRI. Additionally, MRI is costly, not always readily available, and not well‐suited for evaluating joint instability [18]. Compared with other imaging modalities, US is convenient, low‐cost, and radiation‐free and has the potential to become a standard method for diagnosing subtle Lisfranc injuries. However, further research directly comparing the diagnostic accuracy of US, plain radiography, and CT is needed.

Few studies have evaluated Lisfranc ligament injuries using US. One small case series study [23], which compared 10 injured feet with the uninjured contralateral feet, showed a torn dorsal Lisfranc ligament in four patients, with a C1–M2 distance > 2.5 mm. Another cadaveric study assessed whether US could detect Lisfranc injuries with and without abduction stress [5]. The study found that the cutoff value for the C1–M2 articular distance for Lisfranc injuries was 2.04 mm in unstressed US and 2.03 mm in stressed US. The sensitivity, specificity, and area under the curve (AUC) for unstressed US were 47%, 94% and 0.76, respectively, whereas those for stressed US were 81%, 72%, and 0.84, respectively. Although the cutoff values for the C1–M2 articular distance for Lisfranc injuries were similar to the 2.1 mm reported in the present study, the sensitivity and AUC of unstressed US in our study were relatively high. Furthermore, the mean C1–M2 articular distance in unstressed US reported previously was 1.99 ± 0.55 mm [5], which is relatively smaller than the 3.3 ± 1.5 mm reported in the present study. The differences in these results could be attributed to the differences between cadaveric and clinical studies. Subtle Lisfranc injuries in clinical practice are not limited to C1 and M2 injuries but are often accompanied by other injuries, such as transverse‐ or longitudinal‐type injuries [7], which can induce greater instability than simple C1 and M2 injuries [8, 12]. Therefore, although injury patterns were not assessed in this study, several of the cases in the present study may not have been limited to C1 and M2 injuries, resulting in greater instability than that observed in cadaveric C1 and M2 injury models.

This study has several limitations. First, this study only included a small number of patients. However, power analysis showed that 23 feet were sufficient for statistical significance. Second, the indication for surgery was determined by WB plain radiography. Since a previous study has shown that some subtle Lisfranc injuries were missed on WB radiographs [9], WBCT may be a better modality. Thus, our study may be affected by patient selection bias. Nonetheless, because WB plain radiography is a universal method for diagnosing subtle Lisfranc injuries, we believe that the findings of this study are valuable. Third, all surgeons who performed the US examination were highly experienced in this modality. Therefore, the ability of novice US operators to accurately diagnose subtle Lisfranc injury is unclear.

In conclusion, our study demonstrated that US without stress or WB imaging can accurately diagnose subtle Lisfranc injuries, and the intra‐ and inter‐observer reproducibility of the C1–M2 distance measured by US showed perfect agreement. US may be considered a standard method for diagnosing subtle Lisfranc injuries, as it is a convenient, low‐cost, and radiation‐free modality. Nevertheless, further research directly comparing the diagnostic accuracy of US, plain radiography, and CT is warranted.

## AUTHOR CONTRIBUTIONS

*Conceptualisation*: Kensei Yoshimoto and Toru Omodani. *Methodology*: Kensei Yoshimoto and Toru Omodani. *Validation*: Kensei Yoshimoto and Mitsuki Kumaki. *Formal analysis*: Kensei Yoshimoto, Kotaro Ishizuka and Kazunori Maruyama. *Investigation*: Kensei Yoshimoto, Toru Omodani, Kotaro Ishizuka and Kazunori Maruyama. *Resources*: Kensei Yoshimoto. *Data curation*: Kensei Yoshimoto and Mitsuki Kumaki. *Writing—original draft preparation*: Kensei Yoshimoto. *Writing—review and editing*: Kensei Yoshimoto, Toru Omodani, Kazunori Maruyama, Masahiko Noguchi and Ken Okazaki. *Visualisation*: Kensei Yoshimoto. *Supervision*: Masahiko Noguchi and Ken Okazaki. *Project administration*: Kensei Yoshimoto. All authors have read and agreed to the published version of the manuscript.

## CONFLICT OF INTEREST STATEMENT

The authors declare no conflict of interest.

## ETHICS STATEMENT

This study was approved by the institutional review board at Shiseikai Daini Hospital (IRB number: 148). Informed consent was obtained from all patients.

## Data Availability

The data that support the findings of this study are available on request from the corresponding author. The data are not publicly available due to privacy or ethical restrictions.
